# Unveiling the Origin of the Strengthening Mechanism in a Novel Precious Metal Multi‐Principal Elements Alloy

**DOI:** 10.1002/advs.202410936

**Published:** 2024-12-16

**Authors:** Xuan Zhou, Hualong Ge, Kai Xiong, Junjie He, Shunmeng Zhang, Li Fu, Zhilong Tan, Xiaofei Wu, Xuming Li, Haijun Wu, Junmei Guo, Yong Mao

**Affiliations:** ^1^ Materials Genome Institute School of Materials and Energy Yunnan University Kunming 650091 China; ^2^ State Key Laboratory of Advanced Technologies for Comprehensive Utilization of Platinum Metals Yunnan Precious Metals Laboratory Co., Ltd Kunming Institute of Precious Metals Kunming 650106 China

**Keywords:** aging strengthening, chemical short‐range order, nanoindentation, precious metal MPEAs, spinodal decomposition

## Abstract

Precious metal electrical contact materials are pivotal in microelectronic devices due to their excellent chemical stability and electrical properties. Their practical application is hindered by the strength, contact resistance, and high cost. Multi‐principal elements alloys (MPEAs) provide the possibility to develop cost‐effective materials with enhanced mechanical properties. To address this, a novel precious metal MPEA, PdAgCuAuPtZn alloy, is designed, which exhibits significant solid solution strengthening and aging strengthening effects. The ultimate tensile strength increases from 747 MPa in the solution state to 1126 MPa in the aged state, while resistivity remains low. This study presents the first systematic investigation into the strengthening mechanisms of precious metal MPEAs using nanoindentation technology. These findings indicate that the aging strengthening of the alloy is attributed to spinodal decomposition (SD) and chemical short‐range order (CSRO) in the matrix. Furthermore, the precipitation structure with Cu‐rich and Ag‐rich phases gradually replaces the matrix, primarily accounting for aging softening. Additionally, it is discovered that precipitation structure can be strengthened by CSRO formed in the Cu‐rich phase, thus providing an innovative strengthening in PdAgCuAuPtZn alloy. These results will be beneficial to both deeply understanding the aging behaviors of PdAgCuAuPtZn alloys and designing high‐performance precious metal MPEAs.

## Graphical abstract

This study reports a novel precious metal PdAgCuAuPtZn alloy with high strength, low contact resistance, and low cost. Through the solution and aging treatment, this alloy exhibits a significant aging‐strengthening effect and lower resistivity. The aging‐strengthening of this alloy is attributed to spinodal decomposition and chemical short‐range order. These results will be beneficial to designing high‐performance precious metal MPEAs.

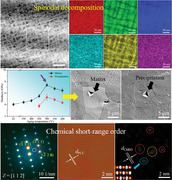

## 1 Introduction

Precious metal alloys have attracted extensive interest due to their excellent chemical stability, superior electrical properties, and good machinability.^[^ ^1^, ^2^, ^3^ ^]^ These alloys hold great promise as electrical contact materials for the aerospace industry and microelectronic devices.^[^ ^4^, ^5^, ^6^ ^]^ In practical applications, the extended service life of these materials necessitates high mechanical properties and low contact resistance. Multi‐principal elements alloys (MPEAs), consisting of at least four principal elements, readily form solid solutions and hold great promise for developing electrical contact materials with excellent properties.^[^ ^7^, ^8^, ^9^ ^]^ Consequently, based on the design concept of MPEAs, precious metal MPEAs have been successfully prepared. At present, fewer articles systematically study the mechanical and resistivity properties of precious metal MPEAs, and most of them only study the mechanical properties but lack the electrical properties.^[^ ^1^, ^2^, ^6^, ^10^, ^11^, ^12^, ^13^, ^14^, ^15^ ^]^ For instance, the PtPdRhIrCuNi alloy, which forms a single‐phase face‐centered cubic (FCC) solid solution, achieves an impressive ultimate compression strength of 1839 MPa.^[^ ^16^ ^]^ Additionally, the IrNiPtRhAl alloy, with an FCC matrix and L1_2_ precipitates, exhibits superior high‐temperature hardness surpassing that of Ni‐based superalloys at temperatures exceeding 900 °C.^[^ ^17^ ^]^ The AuAgCu alloy exhibits lower contact resistance with 11.9 µΩ cm and lower ultimate tensile strength with 745 MPa.^[^ ^18^ ^]^ The novel PtAuCuNi and AuCuNiPd alloys have high ultimate tensile strength with 1497 and 1389  MPa, while these alloys have higher contact resistances with 59.01 and 39.7 µΩ cm.^[^ ^19^, ^20^ ^]^ In summary, achieving a balance between high strength and low contact resistivity in the current research on precious metal electrical contact materials is a significant challenge. Additionally, the high content of precious metals in these alloys can be a disadvantage for cost control. Pd, a more affordable alternative to Au, Pt, and Ir, has significant potential for the development of electrical contact materials that offer a combination of high reliability and hardness. Consequently, the development of novel precious metal electrical contact materials with high strength, low contact resistance, and low cost are crucial for their application.

Many investigations have effectively enhanced the strength of MPEAs via traditional strengthening mechanisms, such as solid solution strengthening, grain boundary strengthening, strain strengthening, and precipitation strengthening.^[^ ^21^, ^22^, ^23^, ^24^ ^]^ As an aging strengthening type of precious metal MPEAs, precipitation during the aging can significantly enhance the strength and hardness of the alloy.^[^ ^12^, ^14^ ^]^ This precipitation, which mainly includes ordered phases and precipitation structure caused by spinodal decomposition (SD), contributes to the increase in strength and decrease in contact resistance. Consequently, precious metal MPEAs can achieve a balance between high strength and low contact resistivity through strong solution strengthening and precipitation strengthening. Recently, it has been found that the enthalpic interactions among constituent elements in MPEAs would lead to local chemical short‐range order (CSRO),^[^ ^25^, ^26^, ^27^, ^28^ ^]^ especially in simple lattice structures such as FCC structure. CSRO refers to different types of atoms that have a preference for ordering on a spatial scale in the first and the next couple of nearest‐neighbor atomic shells around the atom at the center, which leads to a deviation of the atomic arrangement from the ideal random solid solution.^[^ ^29^ ^]^ A few studies have provided direct evidence of CSRO in MPEAs by transmission electron microscopy (TEM).^[^ ^30^, ^31^, ^32^, ^33^ ^]^ Specifically, when the characteristics of CSRO appear, extra diffuse reflections can be observed in selected area electron diffraction (SAED) or nano‐beam electron diffraction (NBED) pattern, usually in the form of a disk, with a diameter substantially larger than that of normal Bragg spots.^[^ ^34^, ^35^ ^]^ However, the key factor in observing extra diffuse reflections is the correct selection of the zone axis. For example, in FCC structures, the extra diffuse reflections due to CSRO are observed along the high‐index zone axis,^[^ ^36^ ^]^ such as [112], while not observed along the low‐index zone axis, such as [110], [111], and [001]. These also provide favorable evidence for us to observe the phenomenon of CSRO.

Therefore, based on the design concept of MPEAs, and considering the properties and cost, the alloy composition with Pd‐30Ag‐14Cu‐10Au‐10Pt‐1Zn (wt.%) has been prepared. In this study, aiming to deeply understand the aging behaviors and increase the properties, combining thermodynamic calculations and experimental validations, the microstructural evolution of PdAgCuAuPtZn alloy during aging treatment was studied, particularly the CSRO and SD, which are limitedly reported in the precious metal MPEAs. We evaluated the correlation between the mechanical and electrical properties and the microstructures. The origin of the aging strengthening was also thoroughly studied by nanoindentation experiments and TEM. This study could provide insights into phase formation, microstructure evolution, and strengthening mechanisms in PdAgCuAuPtZn alloy.

## 2 Results and Discussion

### 2.1 Thermodynamic Calculations

**Figure 1:**
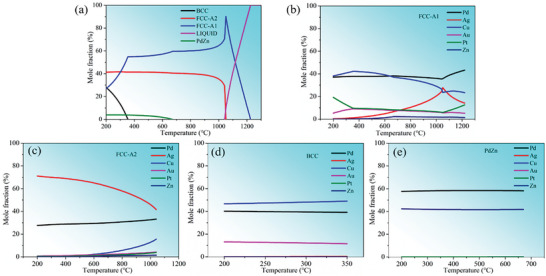
The equilibrium phase transformations and phase composition for PdAgCuAuPtZn alloy as calculated by Thermo‐Calc software.

The equilibrium phase transformations and phase composition were predicted by the calculation of phase diagrams (CALPHAD) method using Thermo‐Calc software (TCNOBL1 database). **Figure** **1** illustrates the predicted equilibrium phase fractions as a function of temperature for the Pd‐30Ag‐14Cu‐10Au‐10Pt‐1Zn alloy. As can be seen, the solidus temperature is ≈1041 °C, and the FCC phase (marked by FCC‐A1) first precipitates as a solid solution in equilibrium with the liquid. Upon further cooling to 1041 °C, another FCC phase (marked by FCC‐A2) precipitates from the supersaturated solid solution, generating in a two‐phase equilibrium region. As the temperature decreases to 670 °C, the PdZn phase commences precipitation, and as the temperature decreases to 350 °C, the BCC phase commences precipitation. The variation in the curves depicted in Figure 1a also indicates that the mole fraction of the FCC‐A1 phase gradually decreases with decreasing temperature, while the mole fraction of the FCC‐A2 phase remains relatively stable. This suggests that the precipitation of the BCC and PdZn phases is associated with the concurrent consumption of the FCC‐A1 phase. Figure 1b–e shows the variation curves of the elemental mole fractions for different phase structures. It can be seen that the FCC‐A2 phase exhibits a higher content of Ag, whereas the content of different elements in the FCC‐A1 phase varies with the change of temperature, among which the content of Cu is higher in the temperature range of 200–600 °C. The BCC phase exhibits a more stable elemental composition, with a consistently higher content of Cu. From Figure 1e, the PdZn phase exhibits a higher content of Pd and Zn. According to the literature, it is known that there are face‐centered tetragonal (FCT) structures between Pd and Zn elements.^[^ ^37^ ^]^ Based on the Thermo‐Calc simulations results, we formulated the solution‐treated temperature of the alloy to be 950 °C, and the aging‐treated temperature to be 350–600 °C.

### 2.2 Mechanical and Electrical Properties

**Figure 2:**
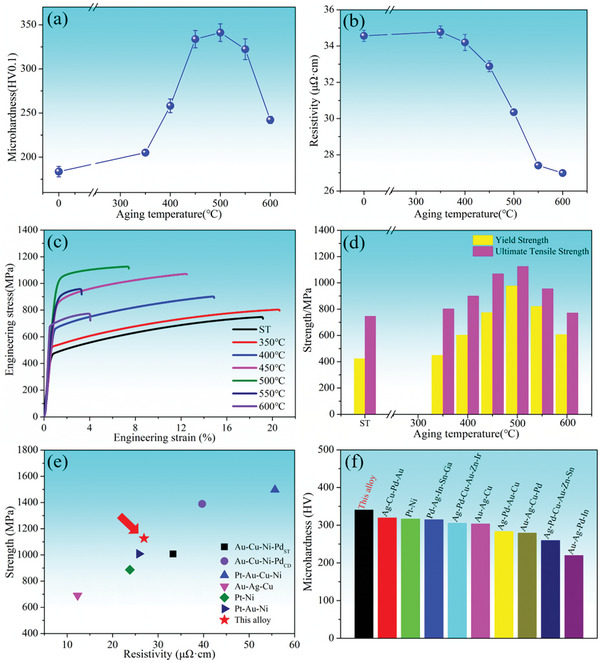
Mechanical and electrical properties. a) Microhardness; b) Resistivity; c) Engineering stress–strain curves; d) Yield strength and ultimate tensile strength values; e) Ultimate tensile strength and as a function of resistivity for this work plotted versus the precious metal MPEAs;^[^ ^18^, ^19^, ^20^, ^27^ ^]^ f) Microhardness for this work versus the precious metal MPEAs.^[^ ^1^, ^2^, ^6^, ^10^, ^11^, ^12^, ^13^, ^14^, ^15^ ^]^ Note these alloys only be studied for microhardness, and not for ultimate tensile strength and resistivity.

**Figure** **2** shows the room‐temperature engineering stress–strain curves, microhardness, and resistivity for the solution‐treated and aging‐treated samples. **Table** **1** shows the mechanical and electrical properties of the alloy. As depicted in Figure 2a, the alloy has a significant aging strengthening effect at the temperature range of 350–600 °C for 1.5 h. In general, this strengthening effect can be divided into two distinct stages. Initially, the microhardness increases with the aging temperature rising from 350 to 500 °C, peaking at a hardness value of 341.1 HV_0.1_ at an aging temperature of 500 °C, improving by 85.8% compared with the solution‐treated sample. Subsequently, the microhardness decreases as the aging temperature rises from 500 to 600 °C, although the values remain higher than those of the solution‐treated sample. The results of yield strength and ultimate tensile strength agree with the microhardness results and the peak values are 978 and 1126 MPa, respectively. Different from the mechanical properties, the resistivity of the alloy consistently decreases with increasing aging temperature, reaching 26.9 µΩ cm at the aging temperature of 600 °C, as shown in Figure 2b, which is also advantageous for its application in electrical contact materials. In summary, the optimum aging process for the alloy is 500 °C/1.5 h. Figure 2e shows the comparison of the tensile strength and resistivity of this alloy with other precious metal MPEAs, and it is worth noting that the mechanical and electrical properties of the other precious metal MPEAs were studied. From Figure 2e, Pd‐30Ag‐14Cu‐10Au‐10Pt‐1Zn alloy achieves the best balance between high strength and low contact resistivity in the current research on precious metal electrical contact materials. Figure 2f shows the comparison of the microhardness of this alloy with other precious metal MPEAs, and it is worth noting that most of them only study the microhardness but lack the electrical properties. From Figure 2f, Pd‐30Ag‐14Cu‐10Au‐10Pt‐1Zn alloy has better microhardness. To deeply understand the aging strengthening mechanism, the microstructures of the alloy after solution treatment and aging treatment were characterized and analyzed.

**Table 1:** The mechanical and electrical properties of the alloy.

Properties	ST	350 °C	400 °C	450 °C	500 °C	550 °C	600 °C
Microhardness, HV_0.1_	183.6	205.2	258.2	333.7	341.1	322.3	242.2
Ultimate tensile strength, MPa	747	803	901	1070	1126	957	773
Yield strength, MPa	424	450	604	775	978	823	607
Elongation, %	19.20	20.60	14.90	12.50	7.40	3.30	4.05
Resistivity, µΩ cm	34.6	34.8	34.2	32.9	30.4	27.4	26.9

### 2.3 Microstructure Evolution

**Figure 3:**
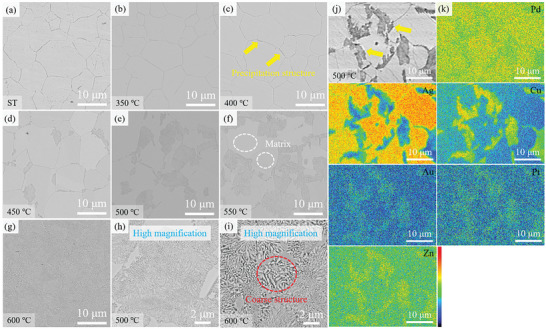
a–g) BSE images of the solution‐treated and aging‐treated samples aged at 350–600 °C; h,i) High magnification of BSE images; j,k) EPMA results showing the elemental distributions of precipitation structure and matrix.

**Figure** **3**a–g shows the backscattered electron (BSE) images of solution‐treated and aging‐treated samples. It can be seen that the solution‐treated sample consists of equiaxed grains with uneven grain sizes from several microns to tens of microns. At an aging temperature of 400 °C, only a small amount of precipitation structure is observed forming at the grain boundaries (indicated by the yellow arrow). Due to the lower temperature, the diffusion of atoms is slower and the growth of precipitation structure is very slower in the early stage of aging treatment.^[^ ^38^ ^]^ As the aging temperature increases from 450 to 500 °C, the precipitation structure at the grain boundaries gradually increases. As the aging temperature rises further, the precipitation structure grows from the grain boundaries to the internal grains and eventually dominates basically the entire matrix. Figure 3h,i show the precipitation structure at higher magnification, revealing that the precipitation structure exhibits a two‐phase morphology of lamellar or short bar. Additionally, the precipitation structure coarsens with increasing aging temperature. The element analysis of precipitation structure and matrix was performed by EPMA, as shown in Figure 3j,k. It can be observed that both Ag and Cu elements show obvious segregation. The precipitation structure mainly consists of Cu‐rich and the matrix mainly consists of Ag‐rich. According to the binary phase diagram of Ag–Cu, Ag, and Cu have solubility limits for each other.^[^ ^11^ ^]^ Pd is distributed across both the precipitation structure and matrix. According to the binary phase diagrams of Pd‐Ag and Pd‐Cu, it is known that the elements Pd and Ag can form a continuous solid solution, and the elements Pd and Cu can form a continuous solid solution but have an ordered structure at low temperatures.^[^ ^39^ ^]^ Combined with the Thermo‐Calc simulations, the alloy is composed of FCC‐A1 and FCC‐A2 as well as PdZn phases at 500 °C, whereas the dual‐FCC phases have a higher content of the Cu and Ag elements, which indicates that the experimental results are in agreement with the results of the Thermo‐Calc simulations.

**Figure 4:**
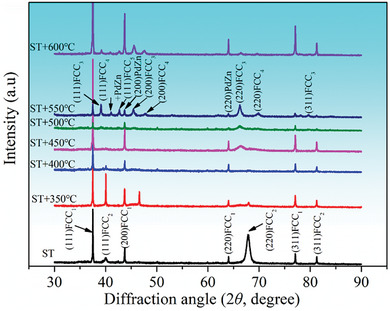
XRD patterns of the solution‐treated and aging‐treated samples.

To investigate the phase transformation during aging treatment, the crystalline structure was determined by XRD, as displayed in **Figure** **4**. From Figure 4, the dual‐FCC phase structure is identified from the XRD patterns in the solution‐treated sample. The lattice parameters are calculated to be 4.039 Å (FCC_1_) and 3.906 Å (FCC_2_), respectively. The elements of Pd, Ag, Cu, Au, and Pt have FCC structures with lattice parameters of 3.923, 4.0863, 3.6148, 4.0786, and 3.923 Å, respectively, and the Zn element has a hexagonal close‐packed structure with lattice parameters of *a = *2.665 Å and *c *= 4.947 Å. Considering the composition of the alloy, the intensity of diffraction peaks, and the lattice parameters obtained from the XRD analysis, it is thought that the parent FCC_1_ is based on the Ag‐rich solid solution containing elements of relatively small atomic size and that the parent FCC_2_ is based on the Cu‐rich solid solution containing elements of relatively large atomic size. This is consistent with the Thermo‐Calc simulated results. As the aging temperature increases, the diffraction peak intensities of the FCC_1_ and FCC_2_ phases gradually decrease and the diffraction peak begins to separate, that is, phase decomposition occurs in the alloy. In the pattern of the sample aged at 550 °C, three FCC peaks (FCC_3_, FCC_4_, FCC_5_) are observed with lattice parameters of 3.970, 3.796, and 3.669 Å, respectively. It is interesting to note that the (111), (200) and (220) diffraction peaks of a FCT structure emerge with a lattice parameter of *a *= 4.135 Å and *c* = 3.398 Å. The reported lattice parameters of the PdZn phase are *a* = 4.1 Å and *c* = 3.346 Å, which are somewhat different from those obtained in the present study. The difference in the lattice parameters is caused by the fact that the PdZn phase in the present study contains other elements besides Pd and Zn, as will be detailed in Section 2.4.

**Figure 5:**
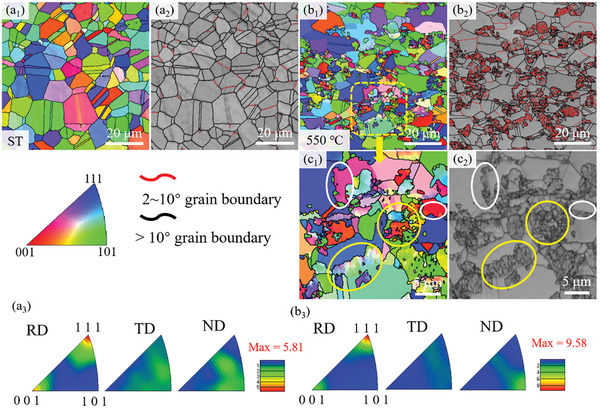
The IPF and BC images. a_1_–a_3_) solution‐treated sample; b_1_–b_3_) aging‐treated sample at 550 °C; c_1_,c_2_) the locally magnified image of the framed region in (b_1_).

The electron back‐scatter diffraction (EBSD) images of crystal orientation inverse pole figure (IPF) and band contrast (BC) are shown in **Figure** **5**. From Figure 5a_1_,b_1_, the average grain size gradually decreases from 4.04 µm in the solution state to 1.07 µm in the aging state at 550 °C, which is mainly related to the precipitation structure at the grain boundaries. Due to the growth of precipitation structure, multiple precipitation structure interfaces contact each other to form colonies, resulting in the segmentation of the matrix into grains with different sizes. From Figure 5a_2_,b_2_, the precipitation structure has low‐angle grain boundaries (LAGBs), indicating that the interfacial orientation difference between the two phases inside the precipitation structure is small, and the adjacent grain has high‐angle grain boundaries (HAGBs). In general, the nucleation driving force for precipitation structure is from the difference between the interface energy and the strain energy of the matrix‐precipitation phase, and the difference determines the formation and growth rate of the precipitation structure. During the growth of the precipitation structure, the redistribution of solutes is not affected by diffusion in the matrix but is determined by the diffusion from the grain boundaries at the interface. Therefore, the HAGBs migration is necessary for the occurrence of precipitation structure.

Further analysis of the grain orientation relationships of the aging‐treated sample revealed that in addition to a small number of annealed twins, the precipitation structures have a different orientation relationship with the adjacent grains, which suggests that the orientation of the two phases has changed after the aging treatment. There are two specific types: 1) The precipitation structure has the same orientation relationship with the adjacent grains, as shown by the white circle in Figure 5c_1_,c_2_. At this time, there is no interface between the precipitation structure and the matrix; 2) The precipitation structure has a different orientation relationship with the adjacent grains, as shown by the yellow circle in Figure 5c_1_,c_2_. From Figure 5a_3_,b_3_, the grain orientation for the solution‐treated sample was dominated by RD direction <111> and <001> stronger textures and TD/ND direction weaker textures. At the aging treatment of 550 °C, the texture distribution is not significantly different, only the texture strength increased from 5.81 to 9.58, indicating that the grain orientation of the aging‐treated sample has not changed compared with the solution‐treated sample.

### 2.4 TEM Characterization

**Figure 6:**
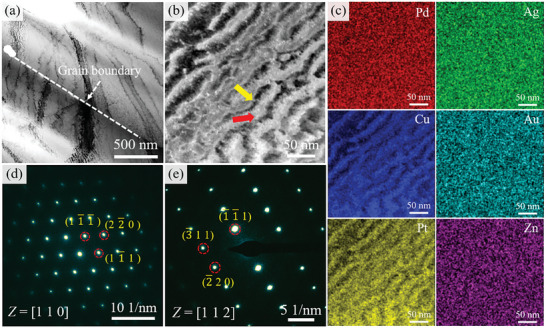
a,d,e) BF image and SAED patterns along the [110] and [112] zone axes of solution‐treated sample; b,c) HAADF and EDS images of solution‐treated sample from clear region showing the compositions of the modulated regions in the microstructure.

Microstructure analysis in Section 2.3 showed that the aging strengthening is speculated to be related to the precipitation structure and matrix. Therefore, the precipitation structure and matrix of the solution‐treated sample and aging‐treated sample were analyzed in detail using TEM. **Figure** **6**a,d,e show the bright field (BF) and corresponding SAED patterns of the solution‐treated sample. From Figure 6a,d,e, the precipitation is not observed at the grain boundary and the SAED results also indicate that the solution‐treated sample has FCC structure. The HAADF image and energy dispersive spectroscopy (EDS) images are shown in Figure 6b,c. The results indicate that two phases evolve into a very fine structure at the nanometer scale in the “clear” grain regions (no precipitation structure).

**Figure 7:**
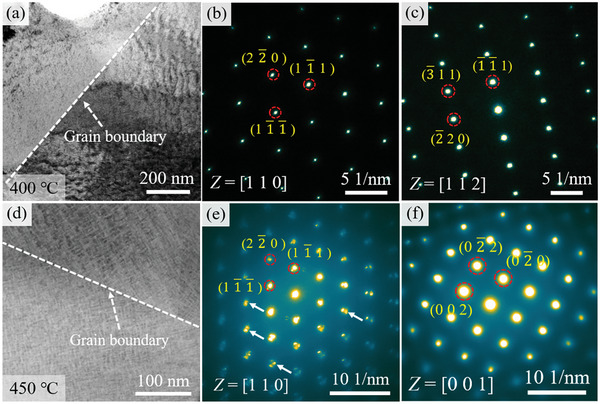
a–c) BF image and SAED patterns along the [110] and [112] zone axes of the sample aged at 400 °C, showing the characteristic feature of the matrix; d–f) HAADF image and SAED patterns along the [110] and [001] zone axes of the sample aged at 450 °C, showing the obvious characteristic feature of SD.

**Figure** **7**a–c shows the TEM results for the sample aged at 400 °C. From Figure 7a, the black nanoparticles appear in the matrix. The SAED patterns along the [110] and [112] zone axes indicate that the sample has an FCC structure. It is speculated that the microstructure occurs in SD, but due to the lower temperature, the elastic stress caused by SD is small, resulting in the absence of obvious diffraction spots in the SAED patterns.^[^ ^40^ ^]^ Figure 7d–f shows the TEM results for the sample aged at 450 °C. It can be seen that the size of black nanoparticles in the matrix increases and the microstructure shows a networked distribution. From Figure 7e, the asymmetric diffraction spots can be observed at the edge of diffraction spots marked by white arrows along the [110] zone axis, which is one of the characteristic features of SD.^[^ ^41^ ^]^ It indicates that the increase in aging temperature led to the occurrence of SD in the matrix.

**Figure 8:**
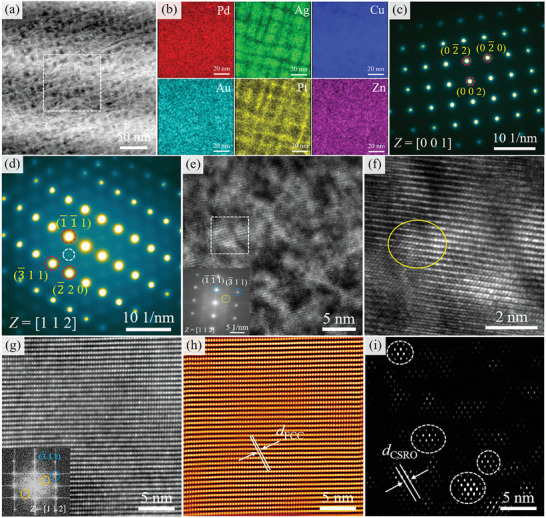
a,b) HAADF and EDS images of the sample aged at 500 °C, showing the obvious characteristic feature of SD; c,d) SAED patterns along the [001] and [112] zone axes. Note the presence of extra diffuse reflections (marked by the white circle) at 1/2($\bar{3}11$) position; e) HRTEM along the [112] zone axis and inset FFT showing the presence of CSRO; f) The locally magnified image of the framed region in (e); g) HAADF lattice image of FCC phase with [112] zone axis. Inset: the corresponding FFT pattern showing the extra diffuse reflections at 1/2($\bar{3}11$) position marked by yellow circles; h,i) Inverse FFT images of FCC lattice and CSRO regions marked by circles, respectively.

**Figure** **8** shows the TEM results for the sample aged at 500 °C. From Figure 8a, the SD structure is obvious with the increase of aging temperature, which indicates that solute elements are further enriched. The energy spectrum analysis of SD in Figure 8b is more obvious and presents the network structure. The modulated structure has a period of ≈15 nm. Figure 8c,d show the SAED patterns along the [001] and [112] zone axes, and it is interesting to note that extra diffuse reflections can be seen at the 1/2($\bar{3}11$) position, as shown by the white circles in Figure 8d. According to previous studies reported, this suggests that the CSRO appears.^[^ ^30^ ^]^ The CSRO structure can be observed from a locally magnified image of High‐Resolution Transmission Electron Microscopy (HRTEM), marked by a yellow circle in Figure 8f. Recent TEM studies on high/medium entropy alloys (H/MEAs) have proposed that the presence of CSRO can be confirmed by observing diffuse spots at 1/2{311} FCC (i.e., halfway between the transmission spot (000) and {311}) in the SAED pattern along the {112} zone axes.^[^ ^36^, ^42^ ^]^ Thus, based on our observations and literature, we attribute the diffuse reflections along {311} to the presence of CSRO. To further observe the phenomenon of CSRO in the matrix, the atomic‐scale HAADF image along the [112] zone axis of the matrix is obtained, as shown in Figure 8g. From the Fast Fourier Transform (FFT) image inset in Figure 8g, the weak extra diffuse reflections between the (000) and ($\bar{3}11$) diffraction spots can be observed. Based on the FFT image, the inverse FFT image is obtained, as shown in Figure 8h,i. Figure 8h shows the FCC lattice image taken using ($\bar{3}11$) diffraction spots. Figure 8i shows the CSRO region taken using extra diffuse reflections and the CSRO region is circled by dashed circles. By comparative analysis, the interplanar spacing (*d*_CSRO_) of CSRO is twice that of FCC interplanar spacing (*d*_FCC)_, which is why extra diffuse reflections appear at the 1/2($\bar{3}11$) position in Figure 8d.

**Figure 9:**
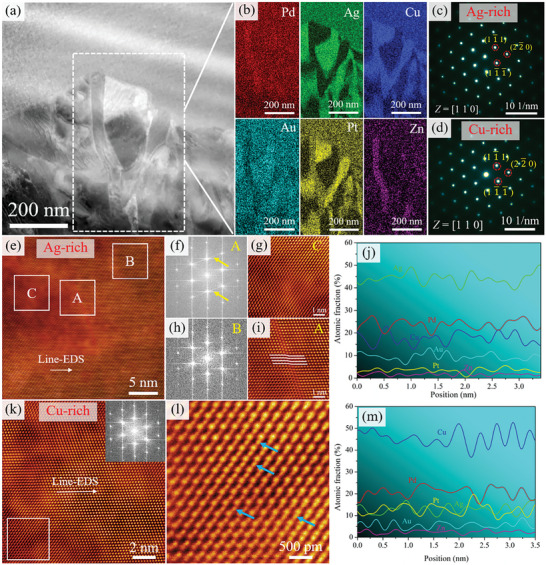
Microstructural observation of precipitation structure at the aging treatment of 500 °C. a,b) HAADF and EDS images; c,d) NBED patterns along the [110] zone axis in Ag‐rich phase and Cu‐rich phase; e) Atomic‐scale HAADF images taken along [110] zone axis in Ag‐rich phase, showing the presence of SF in the matrix; The FFT patterns from A and B regions were shown in (f,h), showing that the matrix is disordered and arrows in (f) indicating the streaking effect of SF; The locally magnified images of A and C regions were shown in (i,g); j) The line‐EDS in (e); k) HAADF image taken along [110] zone axis in Cu‐rich phase; l) The locally magnified images of the framed region in (k), showing that these atomic columns are shifted on the atoms of the matrix; m) The line‐EDS in (k).

**Figure** **9**a–d shows the HAADF and NBED patterns at the grain boundary of the sample aged at 500 °C. It can be seen that the precipitation structure exhibits different morphologies. The EDS results also reveal an obvious segregation of Ag and Cu elements, which is consistent with the EPMA results. Therefore, these two‐phases are defined as the Cu‐rich phase and the Ag‐rich phase. The NBED results along [110] zone axis of the Ag‐rich phase and Cu‐rich phase indicate that the two‐phases have FCC structure, which is consistent with the Thermo‐Calc simulation results. Figure 9e–m shows the atomic‐scale HAADF images and corresponding FFTs along [110] zone axis in the Ag‐rich phase and Cu‐rich phase. From the atomic‐scale HAADF images, the structural inhomogeneity is observed in the Ag‐rich phase and Cu‐rich phase. From Figure 9f, based on the FFT pattern from the A region, we can observe the streaking effect which is usually induced by stacking fault (SF) marked by yellow arrows.^[^ ^43^ ^]^ Figure 9h shows the FFT pattern from the B region. The locally magnified image of A region shows that SF is from the matrix as shown in Figure 9i. In addition to the SF, from the locally magnified image of the C region (Figure 9g), these atomic columns are shifted on the atoms of the matrix, and a similar phenomenon is also observed in the Cu‐rich phase, as shown in Figure 9l marked by blue arrows.

**Figure 10:**
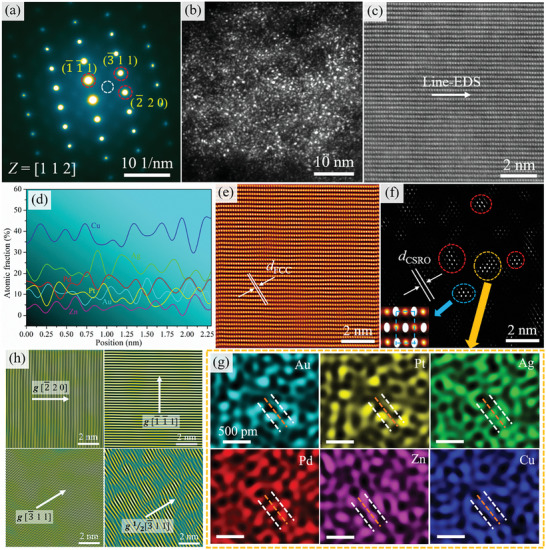
Evidence of CSRO regions in the sample aged at 500 °C. a) The NBED pattern along the [112] zone axis in the Cu‐rich phase. Note the presence of extra diffuse reflections (marked by a white circle) at 1/2($\bar{3}11$) position, showing the existence of CSRO in the Cu‐rich phase; b) Dark‐field TEM image taken using extra diffuse reflections; c) Atomic‐scale HAADF lattice image of FCC phase with [112] zone axis in Cu‐rich phase; d) The line‐EDS in (c); e,f) Inverse FFT images of FCC lattice and CSRO regions marked by circles, respectively. Inset in (f): The close‐up view after superimposing two lattices, both with [112] zone axis. Overlapping this way produces bright sites that highlight the extra CSRO lining up on ($\bar{3}11$) planes; g) The element distribution of CSRO by EDS images with yellow circles in (f), showing that two Au and Pt atoms enrich in the ($\bar{3}11$) plane (white dashed line), while Pd, Ag, Zn, and Cu atoms sandwich between the Au and Pt (brown dashed line), respectively, which are the red, green, pink, and blue dots; h) Inverse FFT fringes corresponding to the FCC planes. Fringes for the ($\bar{2}2$0), ($\bar{1}\bar{1}1$), ($\bar{3}11$), and 1/2($\bar{3}11$) planes along the [112] zone axis. The fringes corresponding to 1/2($\bar{3}11$) are distorted owing to CSRO.

**Figure** **10**a shows the NBED pattern along the [112] zone axis of the Cu‐rich phase and it is interesting to note that the extra diffuse reflections are also observed at 1/2($\bar{3}11$) position in the Cu‐rich phase marked by the white circle. The extra diffuse reflections indicate the existence of a very small‐sized CSRO region in the Cu‐rich phase. The dark‐field (DF) TEM image taken using extra diffuse reflections is shown in Figure 10b, showing the distribution of nanoscale spherical CSRO particles with an average size of ≈0.31 nm. It has been shown before that when CSRO has a length scale of less than 1 nm, it generates a reflection that would expand to a disk ≈5 times the size of a normal reciprocal lattice spot.^[^ ^34^, ^44^ ^]^ The atomic‐scale HAADF image along the [112] zone axis of the Cu‐rich phase shows evidence of CSRO, as shown in Figure 10c. Figure 10d shows the line‐EDS in Figure 10c. Figure 10e shows the FCC lattice image taken using ($\bar{3}11$) diffraction spots. Figure 10f shows the CSRO region taken using extra diffuse reflections and the CSRO region is circled by dashed circles. By comparative analysis, the interplanar spacing (*d*_CSRO_) of CSRO is twice that of FCC interplanar spacing (*d*_FCC_), which is why extra diffuse reflections appear at the 1/2($\bar{3}11$) position in Figure 10a.

After presenting the structure and size of the CSRO, we further probe its chemical composition by EDS mapping at atomic scales. Figure 10g shows one CSRO region (yellow circle in Figure 10f) and close‐up views showing the preferential locations of the six elements in this region. We discover that the CSRO can be best described in terms of Au and Pt occupancy, see the cyan dots in the Au map and yellow dots in the Pt map. Specifically, two Au and Pt atoms enrich in the {311} plane (white dashed line), while Pd, Ag, Zn, and Cu atoms sandwich between the Au and Pt (brown dashed line), respectively, which are the red, green, pink, and blue dots. That is to say that the Au‐enriched and Pt‐enriched {311} planes alternate with those enriched in Pd, Ag, Zn, and Cu. Such a chemical order has a period that doubles the interplanar spacing of the FCC lattice, and explains again why the diffuse reflections appear at the locations corresponding to 1/2($\bar{3}11$). The inverse FFT fringes corresponding to the major FCC planes are presented in Figure 10h. Figure 10h shows the fringes corresponding to the ($\bar{2}2$0), ($\bar{1}\bar{1}1$), ($\bar{3}11$), and 1/2($\bar{3}11$) planes along [112] zone axis, respectively. We observed that the fringes along the 1/2($\bar{3}11$) plane are mostly distorted. These results imply that the presence of CSRO induces lattice distortions.^[^ ^19^, ^20^ ^]^

**Figure 11:**
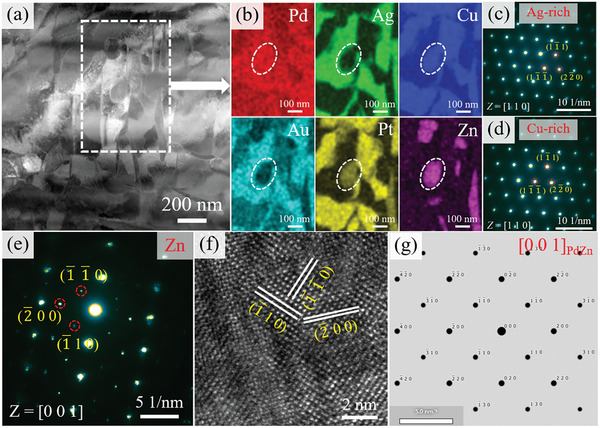
Microstructural observation of precipitation structure in the sample aged at 600 °C. a,b) HAADF and EDS images, showing the elemental distribution of precipitation structure; c,d) NBED patterns of Ag‐rich phase and Cu‐rich phase along the [110] zone axis; e,f) NBED pattern and corresponding HRTEM image of Zn‐rich phase indicating that the Zn‐rich phase has FCT structure; g) Diffraction simulation image along [001] zone axis of PdZn phase.

**Figure** **11** shows the HAADF and EDS images of the precipitation structure in the sample aged at 600 °C. From Figure 11a,b, the precipitation structure is also composed of an Ag‐rich phase and a Cu‐rich phase. Figure 11c,d reveals that the two phases have FCC structures. It is interesting to note that the Zn‐rich phase is primarily composed of Zn, Pd, and Cu elements in the precipitation structure (indicated by dashed lines in Figure 11b. The existence of {110} reflection in the NBED pattern, as depicted in Figure 11e, indicates that the Zn‐rich phase has an FCT structure. This observation is in agreement with the results of the Thermo‐Calc simulation and XRD. Figure 11f shows the corresponding HRTEM image, with the lattice parameter calculated as *a *= 4.246 Å and *c* = 3.484 Å. Figure 11g illustrates the diffraction simulation of the PdZn phase. It was found that the lattice parameter of the Zn‐rich phase differs from that of the PdZn phase, despite the structure being the same. The difference is primarily due to the fact that the Zn‐rich phase in this study is composed not only of Pd and Zn elements but also includes Cu and Pt elements, which explains why the lattice parameter of the Zn‐rich phase differs from the PdZn phase.

### 2.5 Strengthening Mechanism

Spinodal decomposition, with coherent phase boundaries, occurs without any nucleation barrier. In contrast, the formation and coarsening of precipitation structure always must overcome a nucleation barrier, thus requiring higher temperatures and more time to develop in the microstructure. From Figure 7 and Figure 8, SD can be observed in the matrix and became more obvious as the aging temperature increased, and CSRO can be observed in the matrix. The presence of CSRO can increase the strain energy in the localized region, prompting atomic rearrangement to lower the free energy of the system, and thus promote the occurrence of SD. Meanwhile, higher aging temperatures can significantly increase the diffusion rate of alloying elements and promote further enrichment of solute atoms. The inhomogeneous distribution of elements can also be seen in Figure 8b. As the aging temperature increases, the precipitation structure can be observed in the grain boundaries and grows from the grain boundaries to the internal grains. The migrating grain boundary, also known as reaction fronts (RF), acts as a short circuit path for the diffusion of solute atoms, and the concentration difference of solutes across the migrating grain boundary provides the chemical driving force for the growth of precipitation structure. With the growth of the precipitation structure, as the grain boundary migrates into the supersaturated matrix, the solute elements diffuse at the grain boundary, and the results of EPMA confirm the difference in elemental distribution between the precipitation structure and matrix. In MPEA, the inhomogeneous distribution of elements in the SD and precipitation structure is related to the value of enthalpy of mixing (Δ*H*_mix_). Besides that, the pair mixing enthalpies of binary alloys influence the preferential pairing of particular elements, where a negative enthalpy of mixing indicates that the atoms are attracted to each other and a positive enthalpy of mixing indicates that the atoms are repulsed to each other.^[^ ^45^ ^]^ As shown in **Table** **2**, in PdAgCuAuPtZn alloy, the Ag–Cu, Au–Pt, Au–Zn, and Pd–Pt present positive enthalpies of mixing, indicating that atomic bonds are not easily formed between these atoms, and the atomic bonds are preferentially formed in other atoms.

**Table 2:** The Δ*H*_mix_ (kJ mol^−1^) between various elements in PdAgCuAuPtZn alloy.

	Pd	Ag	Cu	Au	Pt	Zn
Pd	–	−7	−14	0	2	−33
Ag	–	–	2	−6	−1	−4
Cu	–	–	–	−32	−12	−4
Au	–	–	–	–	4	12
Pt	–	–	–	–	–	−29
Zn	–	–	–	–	–	–

**Figure 12:**
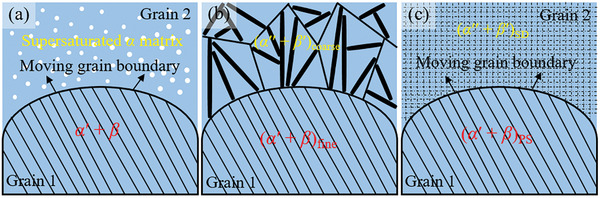
Schematic diagram showing the precipitation reaction of PdAgCuAuPtZn alloy.

The classical precipitation reaction is of the form of *α*→ *α*′ + *β*, as shown in **Figure** **12**a, where the supersaturated *α* matrix transforms behind a migrating grain boundary to a more thermodynamically stable *α* matrix and *β* precipitates.^[^ ^46^ ^]^ In the present study, the precipitation reaction is expressed as *α*_1_ + *α*_2_→ *α*_1_′ + *α*_2_′. As the grain boundaries migrate, the precipitation lamellar structure coarsens and the reaction is expressed as (*α*′ + *β*)_fine_ →(*α*″ + *β*′)_coarse_, as shown in Figure 12b. The driving force for the coarsening of precipitation structure may be only a decrease in surface energy, or it may be chemically driven.^[^ ^47^ ^]^ In the systems that undergo SD prior to precipitation structure, the schematic diagram is shown in Figure 12c. Microstructure analysis revealed that the precipitation reaction occurs in microstructures containing fine‐scale SD. However, the morphologies and transformation mechanisms of the phases formed by the SD and precipitation reactions are completely different, that is, the precipitation reaction is a unique reaction and not merely a coarsening of the SD structure. From the XRD analysis, the solution‐treated sample has dual‐FCC structures, that is, Ag‐rich solid solution and Cu‐rich solid solution. The diffraction peak intensity of Ag‐rich solid solution and Cu‐rich solid solution decreases gradually as the aging temperature increases, especially the Cu‐rich solid solution. The XRD results also show that the diffraction peak occurs to separate, indicating that phase separation is related to the consumption of the Cu‐rich phase. As can also be seen from the results of the Thermo‐Calc simulation, the mole fraction of the FCC‐A1 phase gradually decreases as the temperature decreases, which means that the formation of SD and precipitation structure is related to the consumption of the FCC‐A1 phase. The appearance of different phase structures is attributed to the formation of SD and precipitation structure.

**Figure 13:**
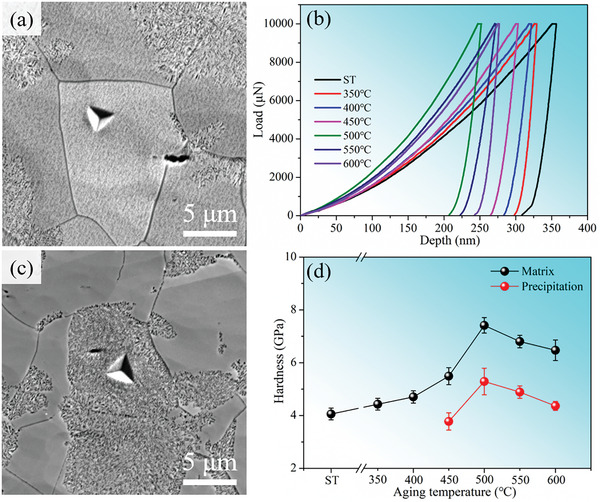
Nanoindentation experiments. a,c) SEM images of the hardness indentations in the matrix and precipitation structure regions; b) Load–displacement curves; d) Hardness of the precipitation structure and matrix.

According to Figure 2, the aging‐treated sample with precipitation structure achieves a great tensile strength–ductility balance compared with the solution‐treated sample. Both microhardness and tensile experiments confirm the appearance of an aging peak at the aging temperature of 500 °C. We further trace the origin of aging strengthening by investigating the temperature dependence of hardness in the precipitation and matrix by nanoindentation. **Figure** **13**a,c show the SEM images of the hardness indentations in the matrix and precipitation structure regions, respectively. Figure 13b shows the load–displacement curves of the matrix in the solution‐treated and aging‐treated samples, respectively. The hardness of the matrix and precipitation structure in the solution‐treated and aging‐treated samples were obtained from the load–displacement curves, as shown in Figure 13d. The harnesses of the matrix and precipitation structure as the function of aging temperature show a trend similar to the yield strength and microhardness, that is, it reaches a maximum at 500 °C and then gradually decreases. The harnesses of the matrix and precipitation structure are 7.42 and 5.29 GPa at the aging temperature of 500 °C. However, when the aging temperature is higher than 500 °C, the microhardness and yield strength gradually decrease, showing the aging softening characteristic. According to the microstructural evolution, the precipitation structure grows from the grain boundary to the internal grain and the spacing of lamellar precipitation structure also increases with the increase of aging temperature, indicating that the precipitation structure occurs coarsening, which has been seen in other alloys as well.^[^ ^38^, ^48^, ^49^ ^]^ The coarsened precipitation structure is probably the main reason for the aging softening. Meanwhile, the nanoindentation results indicate that the hardness of the matrix is higher than that of the precipitation structure. The hardness of the precipitation structure reaches its maximum at 500 °C, which reduces the unfavorable effects on the alloy, and the overall hardness of the alloy is highest at 500 °C.

From the TEM results, the SD structure and CSRO can be observed in the matrix. It is well known that SD structure occurs without any nucleation barrier and periodically distributed in the matrix, resulting in the occurrence of solute‐rich and solute‐lean phases, which generates elastic strain energy that greatly hinders the movement of dislocations. For the TEM analysis of the precipitation structure, the precipitation structure grows from the grain boundary to the internal grain and has dominated basically the entire matrix with aging temperatures ranging from 450 to 600 °C. The EDS results show that the precipitation structure is composed of alternating the Cu‐rich phase and Ag‐rich phase. The extra diffuse reflections can be seen at the 1/2($\bar{3}11$) position in the Cu‐rich phase at the aging treatment of 500 °C. The atomic‐scale HAADF image along the [112] zone axis of the Cu‐rich phase shows evidence of CSRO with an average size of ≈0.31 nm. When the local CSRO regions are uniformly distributed as nanoparticles in the matrix and Cu‐rich phase, they interact with dislocations and hinder the movement of dislocations, thus the alloy could be significantly strengthened. This interaction can be understood as follows: a moving dislocation encounters the field of the ordered region, and an extra force is needed when it encounters and has to break the energetically favored CSRO.^[^ ^29^ ^]^ This entails a trapping effect on the moving dislocations. As a result, the dislocation line migrating through the field of CSRO slows down. This sluggish process is expected to increase the opportunities for dislocations to interact with one another, leading to the occurrence of dislocation pile‐up. Then we can expect the local CSRO regions to increase strain hardening during tensile deformation. The inverse FFT fringes of CSRO in Figure 10h can also be seen as a result of lattice distortion due to the presence of CSRO.

**Figure 14:**
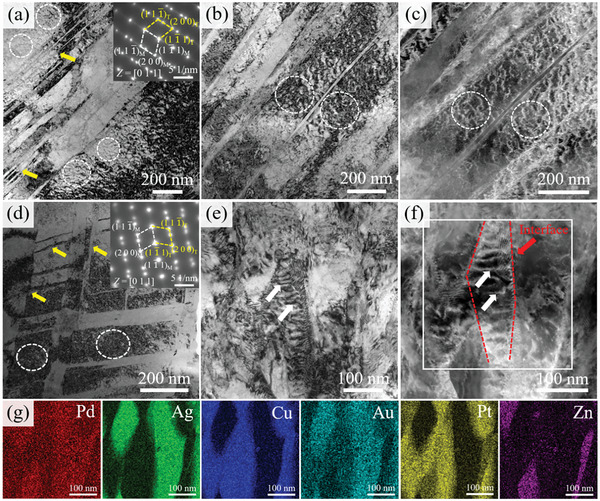
TEM characterization of the deformation structure of the samples aged at 350 °C (a–c) and 500 °C (d–g). a,b) BF images showing the high‐density dislocation and deformation twins. Inset in (a): The SAED pattern of deformation twins. The yellow arrows showing the deformation twins and the white circles showing the dislocation; c) HAADF image corresponding to (b); d,e) BF images showing the high‐density dislocation and deformation twins. Inset in (d): The SAED pattern of deformation twins. The yellow arrows showing the deformation twins and the white circles showing the dislocation; f,g) HAADF and EDS images corresponding to (e). The red line showing the phase interface. The locally magnified maps of the framed region in (f).

To understand the aging strengthening of the alloy, the samples aged at 350 ℃ and 500 °C after tensile fracture were performed by TEM, as shown in **Figure** **14**. Figure 14a–c shows the BF and HAADF images of the samples aged at 350 °C. It can be seen that the deformation twins are observed in the matrix, as shown by the yellow arrows in Figure 14a, and a large number of dislocation pile‐up and dislocation lines appear in deformation twins, as shown by the white circles in Figure 14b,c. Meanwhile, the dislocation lines are observed in the matrix, as shown by the white circles in Figure 14a. Figure 14d–g shows the BF and HAADF images of the samples aged at 500 °C. It can be seen that the deformation twins are also observed in the matrix, as shown by the yellow arrows in Figure 14d. Meanwhile, a large number of dislocations are observed in the matrix, as shown by the white circles in Figure 14d. The difference from the samples aged at 350 °C is that the deformation twins of the samples aged at 500 °C show the cross‐distribution. Deformation twins can offer ample room for dislocation storage, and the generation of multiple twins is more conducive to the work hardening of the alloys. These multiple twins offer adequate pathways for easy glide and cross‐slip of dislocations to accommodate significant plastic deformation.^[^ ^50^ ^]^ As a result, the samples aged at 350 °C have better elongation. From Figure 14a,d, a large number of dislocation pile‐up and dislocation lines appear in deformation twins, indicating that these deformation twins can effectively hinder the movement of dislocations during the tensile process, which improves the strength of the alloy. Further, according to the EDS analysis, these dislocations can be observed mainly in the Cu‐rich lamellar phase and terminate at the interface of the lamellar two‐phases marked by red arrows in Figure 14f, indicating that the interface of the lamellar two‐phases effectively hinders the movement of dislocations, leading to the increasing strength of the alloy.

The reduction of resistivity can be explained by microstructure after aging treatment. Based on Matthiessen's rule, the resistivity is a combination of defects (*ρ*_def_), phonon interactions (*ρ*_pho_), and impurities (*ρ*_imp_):^[^ ^51^ ^]^

(1) $$\rho =\rho_{def}+\rho_{pho}+\rho_{imp}$$

The value of *ρ*_def_ is related to the density of dislocation, interface, vacancies, and grain boundary. The value of *ρ*_pho_ is related to the temperature. The value of *ρ*_imp_ is related to the fraction of solute in a solid solution. For solution‐treated and aging‐treated samples, the value of *ρ*_def_ is not considered because the density of dislocation, interface, vacancies, and grain boundary is insignificant. The value of *ρ*_pho_ for a defect‐free Pd‐30Ag‐14Cu‐10Au‐10Pt‐1Zn alloy is a combination of the resistivity of pure Pd, Ag, Cu, Au, Pt, and Zn, which is insignificant for multi‐principal component alloy.^[^ ^19^, ^20^ ^]^ Therefore, the resistivity of solution‐treated and aging‐treated samples is related to the value of *ρ*_imp_. The solution‐treated sample has a large fraction of solute in the solid solution, resulting in a large lattice distortion and higher *ρ*_imp_. Thus, the solution‐treated sample has high electrical resistivity. After aging treatment, SD and order structure appear in the samples, which reduces the solute atoms in the supersaturated solid solution, and the values of *ρ*_imp_ gradually decrease. It has been reported that SD and ordering will decompose the supersaturated solid solution, reducing the resistivity of Cu‐based alloys.^[^ ^19^ ^]^ Meanwhile, precipitation structure at the grain boundary occurs after aging temperature higher than 400 °C, leading to fewer solute atoms in the aging‐treated samples and lower resistivity. The presence of the precipitation structure increases the phase interface, that is, it increases the scattering of electrons at the interface. However, this effect has relatively little effect on the resistivity because the scattering of electrons at the interface between the precipitation structure and matrix is small. With the increase of aging temperature, the volume fraction of precipitation structure gradually increases. At this time, the interfacial resistivity gradually becomes the main resistivity for aging‐treated samples and it becomes the main reason for the reduction in resistivity of aging‐treated samples.

## 3 Conclusion

In this study, we systematically investigated the microstructural evolution and strength mechanism of PdAgCuAuPtZn alloy during aging treatment using SEM, XRD, EPMA, and TEM. The nano micro‐mechanical properties of PdAgCuAuPtZn alloy were investigated using nanoindentation, revealing the origin of the strengthening mechanism of the alloy. The main conclusions are as follows.

1. Thermo‐Calc simulation elucidated that two‐phase equilibrium regions are composed of a Cu‐rich phase and an Ag‐rich phase at the temperature range of 670–1041 °C. As the temperature decreases, the PdZn phase and BCC phase precipitate. XRD analysis confirms that the solution‐treated sample has a dual‐FCC phase structure, and the diffraction peak begins to separate, indicating phase decomposition occurs in the alloy;
2. The microhardness and yield strength show a tendency to increase first and then decrease with the increase of aging temperature, reaching the aging peak at 500 °C, with values of 341.1 HV_0.1_ and 978 MPa, respectively. The resistivity gradually decreases. Nanoindentation results show that the variation trends of hardness in the precipitation structure and matrix as a function of increasing temperature are similar to those of yield strength and microhardness, reaching 5.29 and 7.42 GPa at 500 °C, respectively.
3. TEM results show that the aging strengthening of the alloy is attributed to the SD and CSRO in the matrix while aging softening is attributed to the coarsening of the precipitation structure. The overall hardness is a comprehensive result of the favorable effects of SD and CSRO on the strengthening of the matrix and the unfavorable effects of precipitation structure on strengthening. TEM analysis of the tensile samples has unveiled the different deformation mechanisms in the PdAgCuAuPtZn alloy. The interface of the Cu‐rich phase and Ag‐rich phase in the precipitation structure can effectively hinder the dislocation movement, and the interaction of deformation twins and dislocations in the matrix contributes to its hardening.
4. The solution‐treated sample has a large fraction of solute in the solid solution, resulting in high resistivity. SD and ordering for the aging‐treated samples will decompose the supersaturated solid solution, which reduces the solute atoms in the supersaturated solid solution, and the resistivity gradually decreases. With the increase of aging temperature, the interfacial resistivity between precipitation structure and matrix becomes the main reason for the reduction in resistivity of aging‐treated samples.

## 4 Experimental Section

### Sample Preparation

Pd, Ag, Cu, Au, Pt, and Zn with high purity (99.99%) were used as raw materials for the alloy, and the raw materials were mixed and ultrasonically cleaned. The as‐cast Pd‐30Ag‐14Cu‐10Au‐10Pt‐1Zn (wt.%) ingot was prepared by high vacuum high‐frequency induction. To improve the microstructural homogeneity of the ingot, the ingot was turned over and re‐melted at least 5 times. The prepared ingot was homogenized to eliminate elemental segregation. First, the ingot was put in a quartz tube. After the vacuum level had reached 10^−3 ^Pa, the quartz tube with the ingot was sealed using a duplex vacuum sealing machine. After, the sealed quartz tubes were put in a muffle furnace and subjected to homogenized annealing at a temperature of 900 °C/6 h. Then, annealed samples were cold rolled into flakes and the flakes were cut into strips using the wire cutting machine. Finally, the striped samples were cold‐drawn to wires with a diameter of 0.4 mm. To study the aging behavior for the Pd‐30Ag‐14Cu‐10Au‐10Pt‐1Zn alloy, the samples were first solution‐treated at 950 °C/1 h, and then aging‐treated in the temperature range of 350–600 °C/1.5 h. Finally, the solution‐treated and aging‐treated samples were mechanically ground, polished, and etched chemically. The etching liquid was freshly prepared by adding 4–5 drops of supersaturated CrO_3_ solution to 10 ml of hydrochloric acid.

### Material Characterization

To identify the equilibrium phase transformation of the alloy, Thermo‐Calc software (TCNOBL1 database) was used for the calculation of thermodynamic equilibrium and phase diagram in the temperature range of 200–1300 °C. The morphologies of solution‐treated and aging‐treated samples were characterized using scanning electron microscopy (TESCAN AMBER). EBSD was carried out by an Oxford Symmetry detector equipped with the above SEM to investigate the grain size and orientation. All the data were analyzed via Channel 5 Software. The EBSD samples were fabricated by Ar ion polishing using a precision ion polishing system (Gatan PECS II 685). The operating angles of the guns are ±5° and the voltage is 7 kV for 1 h. Then, the operating angles are ±3° and the voltage is 5 kV for 1 h. The crystal structure was examined by X‐ray diffraction (XRD, Rigaku Smartlab SE, Cu‐K_α_ radiation, *λ* = 0.15406 nm). The energy spectrum analysis of the matrix and precipitation structure was carried out using an electron probe X‐ray micro‐analyzer (EPMA, JXA‐8230). TEM samples were obtained from the heat‐treated samples through the focused ion beam (FIB) technology. The FIB lift‐out technology involved the selection region, formation of the Pt protective layer, rough milling of the surrounding part, transfer and fixation of thin sections extracted from the bulk to the TEM grid, and a final thinning of thin sections. The detailed microstructure was characterized by TEM (Talos F200X) and atomic‐scale high‐angle annular dark‐field (HAADF) images were obtained by a ThermoFisher Spectra 300 microscope operated 300 kV.

### Properties Testing

The microhardness of solution‐treated and aging‐treated samples was measured by an HVS‐1000Z microhardness tester with a load of 0.98 N and a dwell time of 15 s. Each experimental value represented the average of 10 measurements for each sample. To unveil the origin of the strengthening mechanism of the alloy, nanoindentation experiments were performed on the matrix and precipitation structure using a Hysitron TI 980 Triboindenter, at least 10 indents with a maximum load of 10 mN and a holding time of 5 s. The electrical resistivity for solution‐treated and aging‐treated samples was tested using an AT510PRO resistance tester. Tensile experiments were performed using a Wance EMT 205D Micro computer‐controlled Electromechanical Universal Testing Machine at a tensile rate of 2 mm min^−1^.

## Conflict of Interest

The authors declare no conflict of interest.

## Data Availability

The data that support the findings of this study are available from the corresponding author upon reasonable request.
